# Integrated silicon carbide electro-optic modulator

**DOI:** 10.1038/s41467-022-29448-5

**Published:** 2022-04-05

**Authors:** Keith Powell, Liwei Li, Amirhassan Shams-Ansari, Jianfu Wang, Debin Meng, Neil Sinclair, Jiangdong Deng, Marko Lončar, Xiaoke Yi

**Affiliations:** 1 School of Electrical and Information Engineering, The University of Sydney, Sydney, NSW 2006 Australia; 2 John A. Paulson School of Engineering and Applied Sciences, Harvard University, Cambridge, MA 02138 USA; 3 Division of Physics, Mathematics and Astronomy, and Alliance for Quantum Technologies (AQT), California Institute of Technology, 1200 E. California Boulevard, Pasadena, CA 91125 USA; 4 Center for Nanoscale Systems, Harvard University, Cambridge, MA 02138 USA

**Keywords:** Applied optics, Micro-optics

## Abstract

Owing to its attractive optical and electronic properties, silicon carbide is an emerging platform for integrated photonics. However an integral component of the platform is missing—an electro-optic modulator, a device which encodes electrical signals onto light. As a non-centrosymmetric crystal, silicon carbide exhibits the Pockels effect, yet a modulator has not been realized since the discovery of this effect more than three decades ago. Here we design, fabricate, and demonstrate a Pockels modulator in silicon carbide. Specifically, we realize a waveguide-integrated, small form-factor, gigahertz-bandwidth modulator that operates using complementary metal-oxide-semiconductor (CMOS)-level voltages on a thin film of silicon carbide on insulator. Our device is fabricated using a CMOS foundry compatible fabrication process and features no signal degradation, no presence of photorefractive effects, and stable operation at high optical intensities (913 kW/mm^2^), allowing for high optical signal-to-noise ratios for modern communications. Our work unites Pockels electro-optics with a CMOS foundry compatible platform in silicon carbide.

## Abstract (web summary)

Electro-optic modulator is used to encode electrical signals onto light. Here the authors demonstrate an electro-optic modulator, based on Silicon Carbide, which can be useful for quantum and optical communications.

## Introduction

Being a compelling semiconductor material for next-generation electronic devices^1^ and a contender for realizing integration of electrically-driven quantum emitters^2^, there is motivation to investigate silicon carbide (SiC) for monolithically integrated photonics^2–6^. By taking advantage of the high refractive index (~2.57), wide band-gap, low thermo-optic coefficient^7^, high electron mobility, and thermal conductivity^8^ of SiC, high-density integrated photonic devices can be fabricated in this material with robust properties. Furthermore, this may be accomplished with low fabrication costs due to its compatibility with complementary metal-oxide-semiconductor (CMOS) foundry nanofabrication^9^, which also presents the opportunity for integration with electronics. The high optical damage threshold and bulk Young’s modulus of 450 GPa add to the potential of SiC devices operating in harsh environments.

One critical component for SiC photonics remains outstanding, that is, to demonstrate an electro-optic (EO) modulator using the Pockels (or linear EO) effect. This effect, which exists in non-centrosymmetric crystals such as SiC, allows the refractive index to vary linearly and rapidly in proportion to an applied electric field. Consequently, Pockels-based modulators are exploited to achieve high data rates and microwave-conversion efficiencies without the addition of optical loss^10^ and are foundational to many applications^11^. Despite the discovery of the Pockels effect in bulk cubic SiC (3C-SiC) more than three decades ago^12^, an integrated electro-optic modulator has yet to be realized in SiC for a number of reasons including poor crystal quality and difficulty realizing low-loss waveguides^13–16^. Although 3C-SiC can be grown directly onto a silicon substrate, it has been difficult to obtain high-quality thin films due to crystal defects associated with this approach^7,17,18^. Wafer bonding techniques^19^ and annealing processes^7^ have been explored to address these problems. Yet, the former approach yielded multimode waveguides, while the latter resulted in a high (7 dB/cm) optical loss. These issues undermine a SiC Pockels modulator, specifically one that features single-mode waveguides for stable and high extinction-ratio ring modulators or quantum applications^20^.

Here we design, fabricate and demonstrate a SiC EO modulator. Optical modulation is achieved by electrically driving a microring resonator based on sub-micron-wide 3C-SiC-on-insulator waveguides via the Pockels effect. A microring is chosen to enable a compact device footprint (90 µm^2^) while maintaining high modulation performance at low voltage. The modulator is fabricated with a CMOS foundry compatible process and operates at a transmission rate of up to 15 Gbit/s using CMOS-level drive voltages. Importantly, we reduce the impact of polycrystal grains and waveguide surface roughness compared to previous work to demonstrate ~5.2 dB/cm optical loss using a single-mode waveguide. As a consequence of our work, we measure the Pockels coefficient (1.5 pm/V) of 3C-SiC at an infrared wavelength. Moreover, the modulator is able to operate continuously at high optical intensities of up to 913 kW/mm^2^ without signal degradation or the presence of photorefraction, facilitating low-noise microwave and nonlinear photonics^21^ or potentially parametric frequency conversion of weak fields^22^.

## Results

### Design and fabrication

**Fig. 1:**
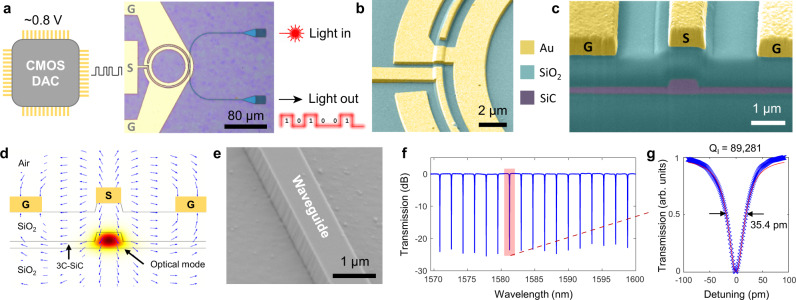
Integrated Pockels modulator in SiC on insulator. **a** Overview of the fabricated ring modulator showing compatibility with CMOS voltages. **b** False color SEM of the microring waveguide and modulator electrodes. **c** False color SEM cross-section of the active region of the modulator. **d** Simulated static electric field and optical mode of the active region of the modulator. **e** SEM of an etched waveguide with the sidewall shown. **f** Measured optical spectrum of the SiC microring resonator after calibrating the optical loss from the VGCs. **g** Lorentz fit of the resonance line shape to determine the intrinsic optical quality factor (*Q*_I_) (Cross: Measurement; Solid line: Lorentz fitting).

The fabrication of the integrated 3C-SiC modulator begins with a 530 nm-thick SiC layer with a low crystal defect density (see Methods) to reduce the scattering or absorption losses and increase EO interaction. Figure 1a shows an optical micrograph of a fabricated modulator capable of being driven by a CMOS digital-to-analog converter (DAC). The modulator consists of a pair of 3C-SiC vertical grating couplers (VGCs) as optical input and output ports, an optical waveguide ring resonator with a loaded quality (Q_L_) factor of 34,310 to balance modulation efficiency and bandwidth^23^, and microwave stripline electrodes to deliver electrical signals. The waveguides and the VGCs are structured by electron beam lithography (EBL) (see Methods), in which a typical waveguide width of 800 nm is chosen to maintain single-mode operation with high optical mode confinement. The electrodes consist of a pair of ground electrodes placed next to the sides of the waveguide and a signal electrode above the waveguide (Fig. 1b). A 1 μm-thick top cladding SiO_2_ layer is deposited to separate the electrode to the waveguide, which prevents optical loss due to mode interaction with the metal. Figure 1c shows a cross-sectional scanning electron micrograph (SEM) of the modulator to illustrate the geometries of the waveguide and electrodes. The electrode thickness (~500 nm) is chosen to reduce radio frequency (RF) loss due to the skin effect. As depicted in Fig. 1d, when a voltage is applied across the signal and ground electrodes, a vertical electric field is induced predominantly in the vertical direction overlapping with the optical mode to probe the Pockels effect. The ring cavity enables the phase change to be translated into an intensity-modulated output, where the resonant enhancement of the modulator allows for a small device footprint and low drive voltage operation. Figure 1e shows an SEM of our etched waveguide, which can achieve a root-mean-squared sidewall roughness l < 2.4 nm facilitating absorption-limited optical loss^7^. To quantify the optical loss, the optical spectrum of the microring resonator with critically coupled resonances operating at the telecommunication wavelengths (1569 nm–1600 nm) is measured to reveal single-mode operation (Fig. 1f) with a resonance linewidth of 35.4 pm (Fig. 1g). The obtained intrinsic Q (Q_I_) is 89,281 corresponding to a linear propagation loss of ~5.2 dB/cm.

### Modulator bandwidth and Pockels coefficient

**Fig. 2:**
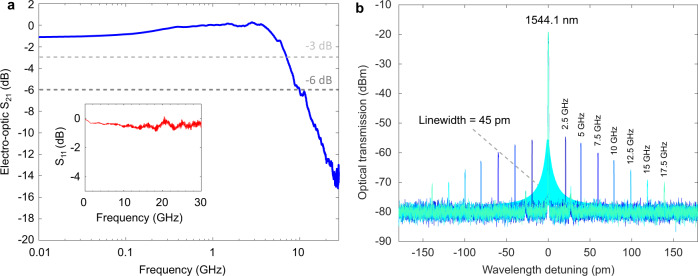
Modulator bandwidth and EO characterization. **a** RF *s*-parameter characterization featuring a −3 dB and −6 dB bandwidths of 7.1 GHz and 9.9 GHz respectively. S21 transmission coefficient of the scattering matrix. Inset shows the S_11_, reflection spectrum of the modulator. **b** Optical spectrum at the output of the modulator for various input RF frequencies. The measurement at 2.5 GHz which is within the resonator linewidth is used in the Pockels coefficient extraction.

To characterize the maximum operational bandwidth of our fabricated modulator, we examine the EO response at an optical input power of 6.8 mW (see Methods), which shows a 3 dB bandwidth of 7.1 GHz (Fig. 2a). The bandwidth is limited by the cavity photon lifetime of 28 ps, calculated based on the measured cavity linewidth (45 pm) that corresponds to the modulation bandwidth of around 5.7 GHz. The electrode circuit of the modulator has a much broader spectral response exceeding 30 GHz, as indicated in the inset of Fig. 2a. Therefore, higher bandwidths could be achieved by reducing the cavity Q factor, however, this will result in a lower modulation index with the same RF signal strength^23^.

To determine the EO performance of the modulator, we use light at a wavelength of 1544.1 nm and drive the modulator with frequencies between 2.5 GHz and 17.5 GHz with a peak-to-peak drive voltage (Vpp) of 1 V (Methods) equivalent to an RF power of 1 mW. The generation of the pair of sidebands seen in the optical spectrum explicitly demonstrates the resultant intensity modulation (Fig. 2b). For increased frequency, a reduction in sideband power is observed, consistent with the roll-off induced by the resonant linewidth. Using the measured optical spectrum and determining the electric field strength inside the waveguide^24,25^, we are able to determine the Pockels coefficient to be 1.5 pm/V (see Methods) which is similar to other CMOS foundry compatible EO materials including AlN (1 pm/V)^25–27^. For a modulation frequency that is less than the resonator linewidth e.g. 2.5 GHz, the modulator can achieve an extinction ratio of 3 dB with Vpp = 8 V. The corresponding half-wave voltage-length product (V_π_L) of SiC on insulator is calculated to be ~55 V·cm (Methods). The combination of a low permittivity (~9.7)^28^ and high refractive index^29^ also allows for more efficient utilization of the linear EO effect in 3C-SiC over other materials, e.g. LiNbO_3_. With direct current (DC) voltages, a resonance shift of 0.11 pm/V (see Methods) is measured, which is lower than the measured RF shift likely due to shielding caused by trapped charges in the silicon-rich SiO_x_ layer^22^. This could be avoided by annealing or using higher purity thermal oxide.

### Digital modulation performance at low drive voltages

**Fig. 3:**
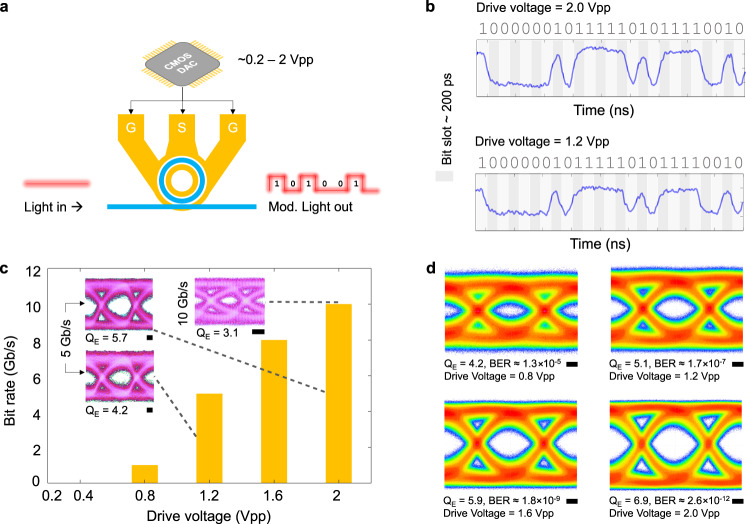
Digital CMOS-level electro-optic modulation with non-return to zero (NRZ) pseudorandom bit sequence (PRBS) of 2^7^ bits. **a** Setup configuration using a CMOS DAC to drive the ground-signal-ground (GSG) electrodes of the modulator. **b** Time-domain waveforms measured at the output of the modulator at 5 Gb/s for drive voltages of 2 Vpp and 1.2 Vpp, respectively. **c** Drive-voltage-dependent eye diagram quality factors (*Q*_E_) for increasing bit rate. The bar plot shows the modulation bandwidth with *Q*_E_ > 2.7. The high-bandwidth photodiode is directly connected to a real-time oscilloscope for recording eye diagrams and *Q*_E_ without equalization. No low-pass RF filter is used in the measurement. **d** Measured eye diagrams and *Q*_E_ at 5 Gbit/s for different drive voltages. A low-pass RF filter is used at the output of the photodiode for inter-symbol-interference reduction. A low-pass RF filter is used at the output of the photodiode for inter-symbol-interference reduction. Eye diagrams and *Q*_E_ are measured via the oscilloscope without equalization. BER is estimated from the measured *Q*_E_ factor^30,31^. Scale bars, 33 ps.

To quantify the performance of our modulator for data transmission, we demonstrate low voltage operation with digital modulation (Methods). Using a non-return-to-zero (NRZ) pseudorandom bit sequence (PRBS) of 2^7^ bits, we drive the modulator directly from a CMOS DAC operating with a *V*pp ranging from 0.2 V to 2 V (Fig. 3a). Figure 3b shows the measured binary data over a period of 5 ns at a data rate of 5 Gb/s, with an optical input power of 6.8 mW using drive voltages of 2 Vpp and 1.2  Vpp, showing that the modulator correctly modulates the light intensity according to the applied digital sequence. Figure 3c depicts the modulator operating at low drive voltages, with an optical input power of 6.8 mW, across a range of modulation bandwidths with an eye *Q*_E_ factor >2.7. When the drive voltage is reduced from *V*pp = 2 V to 1.2 V, the modulator still maintains an open 5 Gb/s non-return-to-zero eye diagram, allowing for successful data transmission and detection. With *V*pp = 2 V and an optical input power of 6.8 mW, the modulator supports bit rates up to 10 Gb/s, limited by the cavity photon lifetime bandwidth (5.7 GHz). The bit error ratio (BER)^30,31^ is obtained from the *Q*_E_ factor of the eye diagram measured by using a low-pass RF filter at the output of the photodetector for inter-symbol-interference reduction (see Methods). The measured eye diagram, Q_E_, and the corresponding BER, at different drive voltages, are shown in Fig. 3d. We obtain a measured *Q*_E_ factor of 4.2 for a drive voltage of 0.8  Vpp, corresponding to a BER of 1.3 × 10^−^^5^. This is lower than the forward error correction (FEC) limit^32,33^.

### High optical power operation

**Fig. 4:**
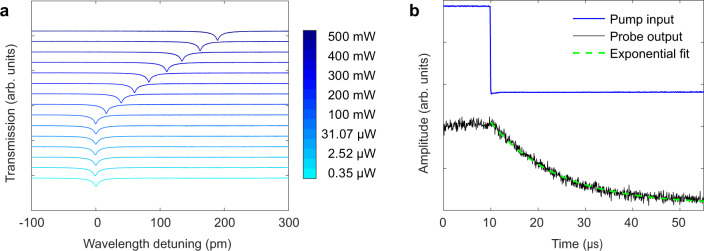
Transmission spectra and relaxation of SiC resonator at high power. **a** Transmission spectra of the probed resonance at 1547 nm with a 1544 nm wavelength pump of varying powers (see colorbar) launching into the VGC to excite the resonator. **b** Step response showing the probe resonance after the pump beam (500 mW power) is switched off. The exponential decay is due to thermal relaxation.

The ability for the modulator to operate at high optical powers without degrading the signal is important for enhancing signal-to-noise ratios in several applications^21,22^. EO materials such as lithium niobate and barium titanate, suffer from signal distortion induced by photorefraction that worsens with increasing optical powers^34–36^, and is avoided by post-modulation amplification in applications. To evaluate the optical response of the modulator at high power, we measure the transmission spectrum of the 3C-SiC modulator while launching different optical pump powers into the VGC using a pump-probe approach (see Methods). Note that the coupling loss of each VGC is around 10 dB for the transverse electric (TE) polarized light used here. The results are displayed in Fig. 4a, showing a redshift of the resonance, where the magnitude of the shift increases linearly with pump power, indicating the expected optothermal effect, which can be minimized via temperature stabilization or cladding optimization^37,38^. The degradation of the optical Q is negligible, even if the input optical power is increased to 500 mW. Moreover, to measure the relaxation of the optical microcavity, we measure the time-dependent change of the resonance by switching off the 500 mW optical pump (see Methods). Figure 4b shows the resonance wavelength relaxes according to a single exponential function. The exponential decay owes to a resonance shift from the cool-down process after the pump power is off^39^, corroborating the redshift of the spectrum observed earlier, further indicating its thermal origin.

**Fig. 5:**
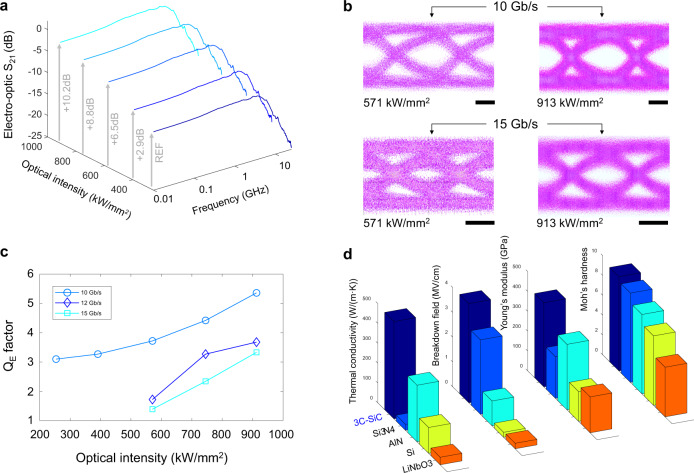
High-power operation and material comparison. **a** Electro-optic s-parameter characterization at high optical intensities shows an improvement in RF responses. **b** Measured eye diagrams at 10 Gb/s and 15 Gb/s. The high-bandwidth photodiode is directly connected to a real-time oscilloscope for recording eye diagrams without equalization. No low-pass RF filter is used in the measurement. Measured eye diagrams confirm increased eye opening and no signal degradation when operating the modulator at high optical intensities. **c** *Q*_E_ factors as a function of optical intensity for bit rates of 10 Gb/s, 12 Gb/s, and 15 Gb/s show an improved and robust modulation performance for higher input intensity. The high-bandwidth photodiode is directly connected to a real-time oscilloscope for recording eye *Q*_E_ factors without equalization. No low-pass RF filter is used in the measurement. **d** Material parameter comparison of 3C-SiC with widely used optical materials showing the distinct advantages of SiC for high power handling. Scale bars, 33 picoseconds.

To quantify operation at high and continuous optical intensities, we measure the EO response of the modulator with varied optical input power. The results are shown in Fig. 5a, in which the optical intensity within the waveguide at the resonance wavelength is calculated from the peak circulating power within the ring resonator^40^. It shows, by increasing the optical intensity from 254 kW/mm^2^ to 913 kW/mm^2^ (corresponding to optical input powers from 6.8 mW to 24.5 mW, respectively), and after accounting for optical insertion loss, the EO response is enhanced by up to 10.2 dB. We observe this without evidence of signal distortion or saturation of modulation efficiency, with the limiting factor being the fiber damage threshold. The observed eye diagrams (Fig. 5b) also exhibit enlarged openings at 10 Gb/s and 15 Gb/s with increased optical intensities. This is due to the increase in signal-to-noise ratio at a higher optical power, which consequently improves the ability to transmit data at a higher speed. High-power operation thus shows improved modulation performance for digital signals and the ability to reach larger bandwidths. Moreover, Fig. 5c shows at an optical intensity of 913 kW/mm^2^ and without equalization and low-pass filtering, *Q*_E_ > 2.7 for all data rates at *V*pp = 2 V. This confirms the operation of the modulator at high optical intensities.

To distinguish SiC for optical and electrical integration, we compare the material parameters of several EO modulator platforms (Fig. 5d). The large thermal conductivity of 3C-SiC (490 W/(m·K)), which is almost double that of AlN^41^ and more than 12-fold larger than LiNbO_3_^42^ benefits SiC for high-power EO applications and co-integration with electronics. Furthermore, the ultra-high breakdown field of 3C-SiC (4 MV/cm), which is 18-fold higher than LiNbO_3_, enables the possibility to integrate RF amplifiers on chip with the modulator as well as making it resistant to electro-magnetic attacks from RF bursts. Moreover, the high radiation hardness, together with the high Moh’s hardness and large Young’s modulus of SiC also present advantages in harsh operating environments^4^.

## Discussion

We demonstrate an integrated Pockels modulator in SiC with a CMOS compatible drive voltage, small device footprint, and continuous low-noise data modulation at high optical intensities. We envision a future device that could achieve improved linearity, spur-free dynamic range, and frequency response by implementing a SiC Mach–Zehnder modulator. Our SiC EO modulator opens opportunities for direct SiC optoelectronic integration using CMOS foundries with the benefit to various applications ranging from optical networks, chip-scale interconnects, RF and microwave photonics, and quantum photonics. Our work constitutes an essential component of an envisioned thin-film SiC platform consisting of monolithic integration of modulators, photodiodes, frequency converters, and quantum functionalities for on-chip photonics. Finally, the ability to integrate with electronics could inspire a generation of integrated devices for photonic signal processing, chip-to-chip, or intra-chip interconnects, thereby ushering in an era of scalable optoelectronics.

## Methods

### Device fabrication

Devices are fabricated from a commercially available 3C-SiC on a silicon wafer with 3.5 μm of SiC epitaxially grown on a silicon substrate supplied by NOVASiC. A 2 μm-thick low pressure chemical vapor deposition (LPCVD) SiO_2_ is deposited onto the SiC thin film, and the resultant stack is Van der Waals bonded to a thermal SiO_2_ on silicon wafer before thinning down SiC via an inductively coupled plasma reactive ion etching (ICP-RIE) process. The device consists of a thin 3C-SiC layer, LPCVD SiO_2_, and a silicon substrate for the handle. The waveguides and grating couplers are patterned on 2 μm of hydrogen silsesquioxane (FOX-16) resist using EBL. They are subsequently etched into the SiC layer using an ICP-RIE process consisting of the CMOS foundry compatible gases SF_6_ and C_4_F_8_^7^. The built-up polymer due to C_4_F_8_ etch gas is removed using a two-step wet cleaning process. First, a solution of hydrogen peroxide and ammonium hydroxide is used to remove the polymer. Second, a solution of hydrogen peroxide and hydrochloric acid is used to remove the metal ions from the surface of the etched waveguides. Plasma enhanced chemical vapor deposition (PECVD) process is used to deposit a 1 μm layer of SiO_2_ onto the fabricated devices to act as an insulation layer between the electrodes and the device, which is sufficiently thick to minimize excess absorption due to the metal electrodes. Device electrodes fabrication involves EBL patterning on Polymethyl methacrylate resist, developed with a mixed solution of one part Methyl isobutyl ketone and three parts Isopropyl alcohol. Metal layers consisting of 5 nm titanium and 500 nm of gold are deposited using electron beam evaporation followed by lift-off using a solution of N-Methyl-2-pyrrolidone.

### Electro-optic characterization and transmission spectrum measurement

Laser light (Keysight 81960 A) around 1550 nm is amplified using an Amonics erbium-doped fiber amplifier (EDFA) followed by an optical bandpass filter to reduce amplified spontaneous emission noise. The resultant light is launched into the ring modulator (Supplementary Fig. 1a). The laser is tuned to a wavelength that matches the most linear edge of a resonance to ensure minimal distortion in the modulated signal. A second EDFA is placed after the modulator to compensate for optical loss before the optical to electrical conversion via a 20 GHz photodetector (Discovery). A high-speed microwave probe (GGB) is used to deliver the modulation signal to the input port of the transmission line. To measure the EO response, the sinusoidal signal with sweeping frequency from a signal generator of a vector network analyzer (VNA, Keysight N5234A) is used to drive the modulator via the microwave probe, while the photodetector output is connected to the VNA receiver. EO response is obtained from the s-parameter of the VNA, where RF cable losses are calibrated out of the measured frequency responses. To measure the high-speed data modulation, electrical PRBS signals of voltage varied from 0.2 Vpp to 2 Vpp are generated from a 65 GSa/s arbitrary waveform generator (AWG, Keysight M8195A) and then connected with the microwave probe (Supplementary Fig. 1b). A real-time digital sampling oscilloscope with an analog bandwidth of 110 GHz (Keysight UXR series) is used to capture the received signal from the photodetector. The oscilloscope acts as the receiver for the PRBS, where a second-order phase-locked loop is used for clock recovery to generate an eye diagram of the received data. The eye *Q*_E_ factor was measured directly from the oscilloscope using persistence mode for a fixed number of waveforms.

The BER^30,31^ is obtained from the eye *Q*_E_ factor measured by using a low-pass RF filter (MiniCircuits VLF-6400+) at the output of the photodetector to reduce inter-symbol interference. The signal from the output of the low-pass RF filter is captured by a real-time digital sampling oscilloscope with an analog bandwidth of 70 GHz (Tektronix DPO77002SX). Eye diagrams and *Q*_E_ are measured via the oscilloscope without equalization.

By scanning the wavelength of the tunable laser and detecting the optical power (Keysight N7744A) at the optical output of the modulator, the transmission spectra for different DC voltages are obtained. DC bias voltages from −20 V to +20 V are applied on the modulator ground-signal-ground electrodes and the transmission spectra graphed (Supplementary Fig. 2).

### Pockels coefficient extraction

Finite element method solver (COMSOL Multiphysics) is used to simulate the optical mode profile and the electric field distribution inside the SiC waveguide. The electro-optic overlap integral^24,25^ (Γ) is numerically calculated to be 0.184 to evaluate the interaction of optical and electric fields based on the measured waveguide and electrode geometries shown in Fig. 1c. The effective refractive index (*n*_eff_) of the fundamental TE mode within the SiC on insulator waveguide at the operating wavelength is 2.27. The modulation index is determined using the Jacobi-Anger expansion method which is obtained from the optical power ratio of the modulated sideband and the optical carrier. The voltage-induced effective refractive index change of the fundamental TE mode is calculated to be 1.37 × 10^−6^/V from the measured resonance shift, and then the EO coefficient (*r*) of the 3C-SiC waveguide at the operating wavelength is derived correspondingly^43^. Considering the refractive index (*n*) of SiC is 2.57^29^ and the electrode gap (g) is 1.9 µm in the experiment, the half-wave voltage-length product (V_π_L) at the operating wavelength (*λ*_0_) of 1544 nm is calculated to be ~55 V·cm, where V_π_L = *n*_eff_*λ*_0_g/(n^4^rΓ).

### Pump-probe measurement

Frequency domain analysis: Light (pump) from a tunable laser (Keysight 81950 A) is amplified by an EDFA to increase the optical power and then coupled into the 3C-SiC modulator placed on a heat sink, where the pump is aligned to the optical resonance of the modulator (1544 nm wavelength). A low-power probe beam, which is synchronized with an optical spectrum analyzer (AP2083A), is used to monitor the transmission spectrum using a resonance that is about ten FSRs red-detuned from the pump (Supplementary Fig. 3). The probe power that is launched into the modulator has a power of <10 µW such that it introduces negligible nonlinear optical effects. Time-domain analysis: We use an optical switch (rise time 1 μs) to switch off the 500 mW power pump beam while keeping the probe beam on. We observe a shift of the cavity resonance at the probe wavelength that follows an exponential dependence owing to the cooling of the resonator. The probe light is tuned to the side of fringe of the optical resonance so that the resonance wavelength change results in the variation of optical power detected by a photodetector connected to an oscilloscope.

### Material parameter comparison

Supplementary Table 1 lists the comparison of common photonic integration materials with 3C-SiC, specifically parameters related to power handling of the modulator. The Young’s modulus and Moh’s hardness are useful parameters for a broad range of applications. Moreover, the refractive index and electrical permittivity are also listed. Low electrical permittivity combined with a higher refractive index is favorable for a larger modulation efficiency at a given EO coefficient.

## Supplementary information

Supplementary Information

## Data Availability

Supplementary Information is available in the online version of the paper. Source data are provided with this paper and also available on Zenodo: https://zenodo.org/record/6342493.
