# Supplementary material for: Integrated silicon carbide electro-optic modulator
Source: Nat Commun. 2022 Apr 5;13:1851. doi: 10.1038/s41467-022-29448-5 (PMC8983721; doi:10.1038/s41467-022-29448-5)
Supplement: Supplementary file 1 — Supplementary Information [file 41467_2022_29448_MOESM1_ESM.pdf]

Supplementary information for:

Integrated silicon carbide electro-optic modulator

Authors:
Keith Powell[1,2], Liwei Li[1], Amirhassan Shams-Ansari[2], Jianfu Wang[1], Debin Meng[1], Neil Sinclair[2,3], Jiangdong Deng[4], Marko Lončar[2*] & Xiaoke Yi[1*]

Author affiliations:
[1]School of Electrical and Information Engineering, the University of Sydney, NSW 2006, Australia.
[2]John A. Paulson School of Engineering and Applied Sciences, Harvard University, Cambridge, MA 02138, USA.
[3]Division of Physics, Mathematics and Astronomy, and Alliance for Quantum Technologies (AQT), California Institute of Technology, 1200 E. California Boulevard, Pasadena, CA 91125, USA.
[4]Center for Nanoscale Systems, Harvard University, Cambridge, MA 02138, USA.

*corresponding author: loncar@seas.harvard.edu; xiaoke.yi@sydney.edu.au

Supplementary Figure 1: Experiment configurations for high-bandwidth measurements.
Supplementary Figure 2: Optical characterization of the electro-optic ring modulator.
Supplementary Figure 3: Experimental setup for optical characterization at high power.
Supplementary Table 1: Comparison of photonic integration platforms for power handling and robustness.
Supplementary References

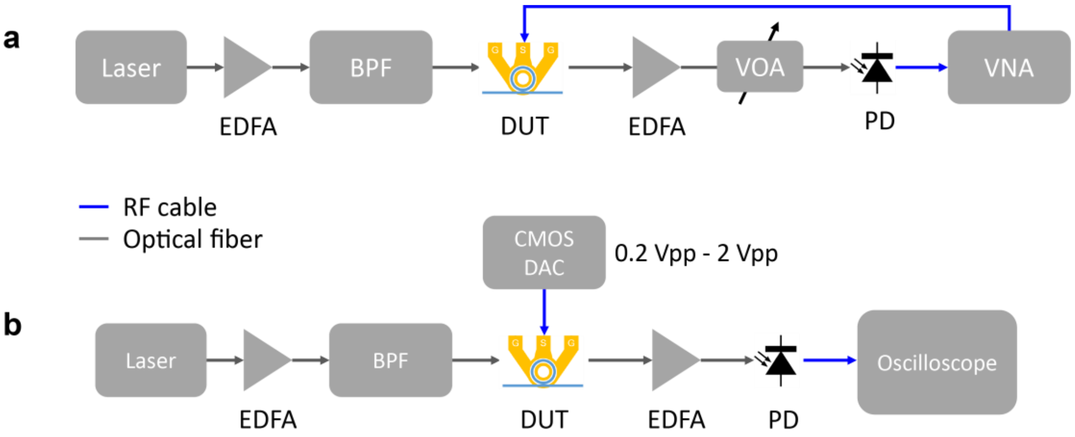

Supplementary Figure 1. Experiment configurations for high-bandwidth measurements. a Setup for measuring the electro-optic response of the SiC modulator. **b** Setup for testing the digital communications operation of the SiC modulator. EDFA, erbium doped amplifier; BPF, bandpass filter; DUT, device under test; VOA, variable optical attenuator; PD, photodiode; VNA, vector network analyzer.

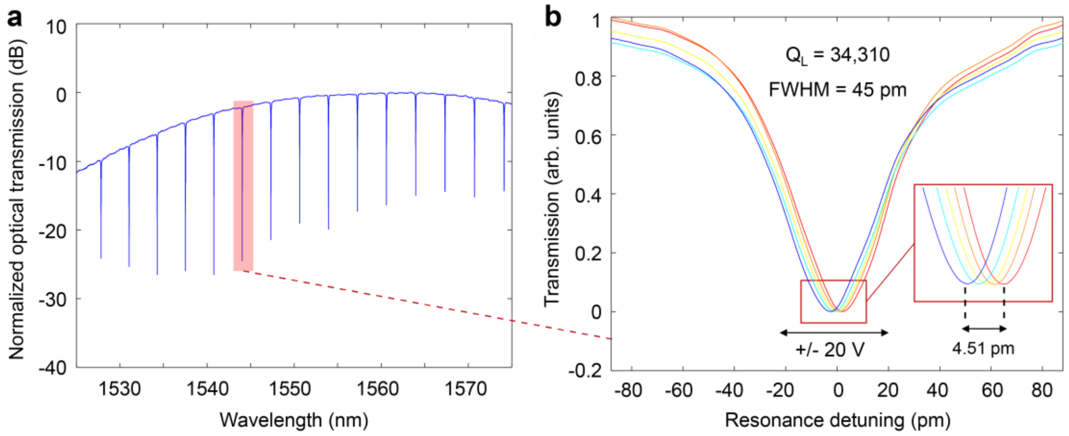

Supplementary Figure 2. Optical characterization of the electro-optic ring modulator. a Optical transmission spectrum showing single mode operation. **b** Measured DC electro-optic resonance detuning and quality (Q) factor of the modulator ring resonator over a DC voltage range of +/- 20 V. FWHM, full-width-half-maximum.

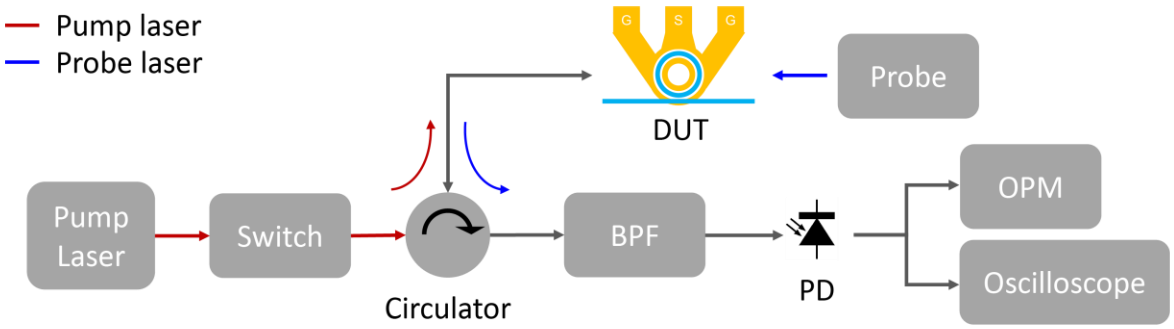

Supplementary Figure 3. Experimental setup for optical characterization at high power. DUT, device under test; BPF, bandpass filter; PD, photodiode; OSA, optical spectrum analyzer.

Supplementary Table 1. | Comparison of photonic integration platforms for power handling and robustness. The large thermal conductivity benefits SiC for high-power EO applications and co-integration with electronics; the ultra-high breakdown field enables the possibility to integrate RF amplifiers on-chip with the modulator as well as making it resistant to electro-magnetic attacks from RF bursts; the high radiation hardness, together with the high Moh's hardness and large Young's modulus of SiC present advantages in harsh operating environments.

	**3C-SiC**	**$LiNbO_3$**	**AlN**	**Si**	**Si_3N_4**
**Relative permittivity**	9.66 [1]	28 [2]	10 [3]	11.8 [1]	5.5 [4]
**Refractive Index @ 1550 nm**	2.57 [5]	n_o = 2.21 [6]<br>n_e = 2.14	2.12 [7]	3.48 [8]	1.98 [9]
**Moh's Hardness**	9.5 [10]	5 [11]	8 [12]	7 [13]	9 [14]
**Breakdown voltage**	4 MV/cm [15]	220 kV/cm [16]	1 MV/cm [7]	200 kV/cm [1]	3 MV/cm [17]
**Thermal conductivity**	490 W/(m·K) [1]	38 W/(m·K) [18]	285 W/(m·K) [7]	130 W/(m·K) [7]	30 W/(m·K) [19]
**Thermo-optic coefficient**	5.8×10^{-5} K^{-1} [20]	3.9×10^{-5} K^{-1} [21]	2.3×10^{-5} K^{-1} [22]	1.8×10^{-4} K^{-1} [23]	2.5×10^{-5} K^{-1} [19]
**Mechanical elastic/Young's modulus**	424 GPa [24]	181 GPa [25]	330 GPa [26]	150 GPa [27]	210 GPa [28]

Supplementary References

1. Bhatnagar, M. & Baliga, B. J. Comparison of 6H-SiC, 3C-SiC, and Si for power devices. *IEEE Trans. Electron Devices* **40**, 645–655 (1993).

2. Guarino, A., Poberaj, G., Rezzonico, D., Degl'Innocenti, R. & Günter, P. Electro-optically tunable microring resonators in lithium niobate. *Nat. Photonics* **1**, 407–410 (2007).

3. Zhu, S. & Lo, G.-Q. Aluminum nitride electro-optic phase shifter for backend integration on silicon. *Opt. Express* **24**, 12501–12506 (2016).

4. Gao, Y., Tao, L., Tsang, H. K. & Shu, C. Graphene-on-silicon nitride waveguide photodetector with interdigital contacts. *Appl. Phys. Lett.* **112**, 211107 (2018).

5. Tropf, W. J., Thomas, M. E. & Linevsky, M. J. Infrared refractive indices and thermo-optic coefficients for several materials. *Optical Diagnostic Methods for Inorganic Transmissive Materials.* **3425**, 160–171 (1998).

6. Lee, Y. S. *et al.* Hybrid Si-$LiNbO_3$ microring electro-optically tunable resonators for active photonic devices. *Opt. Lett.* **36**, 1119-1121 (2011).
7. Xiong, C., Pernice, W. H. P. & Tang, H. X. Low-loss, silicon integrated, aluminum nitride photonic circuits and their use for electro-optic signal processing. *Nano Lett.* **12**, 3562–3568 (2012).
8. Li, H. H. Refractive index of silicon and germanium and its wavelength and temperature derivatives. *J. Phys. Chem. Ref. Data* **9**, 561–658 (1980).
9. Arbabi, A. & Goddard, L. L. Measurements of the refractive indices and thermo-optic coefficients of Si_3N_4 and SiO_X using microring resonances. *Opt. Lett.* **38**, 3878–3881 (2013).
10. Goel, S., Luo, X., Reuben, R. L. & Rashid, W. Bin. Atomistic aspects of ductile responses of cubic silicon carbide during nanometric cutting. *Nanoscale Res. Lett.* **6**, 589 (2011).
11. Arizmendi, L. Photonic applications of lithium niobate crystals. in *Physica Status Solidi (A) Applied Research* **201** 253–283 (2004).
12. Yushkov, Y., Oks, E., Zolotukhin, D. & Tyunkov, A. Properties of thermo-conductive ceramic-based coatings deposited using fore-vacuum plasma-cathode electron source. *7th International Congress on Energy Fluxes and Radiation Effects (EFRE)* 844–847 (2020).
13. Wang, D., Wang, Z., Wang, Z., Qian, G. & Gong, X. Study on hydrocyclone separation enhancement of micro Si/SiC from silicon-sawing waste by selective comminution. *Sep. Sci. Technol.* 56, 991–999 (2020).
14. Moskalewicz, T. *et al.* Electrophoretic deposition and microstructure development of Si_3N_4/polyetheretherketone coatings on titanium alloy. *Surf. Coatings Technol.* **350**, 633–647 (2018).
15. Weitzel, C. E. *et al.* Silicon carbide high-power devices. *IEEE Trans. Electron Devices* **43**, 1732–1741 (1996).
16. Stanicki, B. J. *et al.* Surface domain engineering in lithium niobate. *OSA Contin.* **3**, 345 (2020).
17. Lu, X., Moille, G., Rao, A., Westly, D. A. & Srinivasan, K. Efficient photoinduced second-harmonic generation in silicon nitride photonics. *Nat. Photonics* **15**, 131–136 (2020).
18. Liu, X. *et al.* Highly efficient Thermo-optic Tunable micro-ring resonator based on thin film lithium niobate platform. *Opt. Lett.* **45**, 6318–6321 (2020).
19. Qiu, C. *et al.* All-optical control of light on a graphene-on-silicon nitride chip using thermo-optic effect. *Sci. Rep.* **7**, 17046 (2017).
20. Powell, K. *et al.* High-Q suspended optical resonators in 3C silicon carbide obtained by thermal annealing. *Opt. Express* **28**, 4938–4949 (2020).

21. Wang, C. *et al.* Nanophotonic lithium niobate electro-optic modulators. *Opt. Express* **26**, 1547–1555 (2018).

22. Watanabe, N., Kimoto, T. & Suda, J. The temperature dependence of the refractive indices of GaN and AlN from room temperature up to 515 °C. *J. Appl. Phys.* **104**, 106101 (2008).

23. Komma, J., Schwarz, C., Hofmann, G., Heinert, D. & Nawrodt, R. Thermo-optic coefficient of silicon at 1550 nm and cryogenic temperatures. *Appl. Phys. Lett.* **101**, 041905 (2012).

24. Jackson, K. M., Dunning, J., Zorman, C. A., Mehregany, M. & Sharpe, W. N. Mechanical properties of epitaxial 3C silicon carbide thin films. *J. Microelectromechanical Syst.* **14**, 664–672 (2005).

25. Jiang, W. C. & Lin, Q. Chip-scale cavity optomechanics in lithium niobate. *Sci. Rep.* **6**, 36920 (2016).

26. Xiong, C. *et al.* Aluminum nitride as a new material for chip-scale optomechanics and nonlinear optics. *New J. Phys.* **14**, 095014 (2012).

27. Han, S., Seok, T. J., Quack, N., Yoo, B.-W. & Wu, M. C. Large-scale silicon photonic switches with movable directional couplers. *Optica* **2**, 370–375 (2015).

28. Xiong, W. *et al.* SiNx films and membranes for photonic and MEMS applications. *J. Mater. Sci. Mater. Electron.* **31**, 90–97 (2020).
